# An Approach to Transfer Methods from HPLC to UHPLC Techniques in Some Carbapenems

**DOI:** 10.1007/s10337-014-2725-6

**Published:** 2014-08-17

**Authors:** Przemysław Zalewski, Alicja Talaczyńska, Patrycja Korban, Piotr Garbacki, Mikołaj Mizera, Judyta Cielecka-Piontek

**Affiliations:** Department of Pharmaceutical Chemistry, Faculty of Pharmacy, Poznan University of Medical Sciences, Grunwaldzka 6, 60-780 Poznań, Poland

**Keywords:** Column liquid chromatography, Stability-indicating method, Doripenem, Meropenem, Tebipenem

## Abstract

Stability-indicating LC methods were developed and validated for the quantitative determination of doripenem, meropenem and tebipenem in the presence of their degradation products formed during forced degradation studies. Isocratic HPLC and UHPLC separations were performed with a core–shell Kinetex 1.7, 2.6 and 5 µm, all C18, 100A, 100 × 2.1 mm columns and the mobile phase composed of acetonitrile and 12 mmol L^−1^ ammonium acetate in different ratios. The flow rates of the mobile phase were: 0.5 mL min^−1^ for 1.7 µm column, and 1.0 mL min^−1^ for 2.6 and 5 µm ones. Detection wavelength was 298 nm and temperature was set at 30 °C. All analysed drugs were exposed to stress conditions which caused their hydrolysis and thermal degradation. The methods were validated by evaluation of linearity, accuracy, precision, selectivity and robustness. Proposed methods were successfully applied for the determination of investigated antibiotics during kinetic studies in aqueous solutions and in the solid state. The advantages of chromatographic procedures which are based on the use of C18 stationary phases with different particle sizes in the analysis of selected carbapenems were discussed.

## Introduction

Significant susceptibility of some drugs to degradation requires proper selection of conditions of chromatographic separation. During the optimization of chromatographic procedure, the following features should be taken into account: limitation of reagents, which can catalyze degradation of labile analytes, short run time and achievement of required validation parameters. Among all validation parameters, the most essential is achievement of selectivity [1]. The achievement of suitable selectivity is critical parameter during method validation when the kind of degradation products depends on the affecting factors. Carbapenems doripenem (DOR), meropenem (MER) and tebipenem (TEB) were chosen as model drugs, which exhibit a significant instability in aqueous solutions and in the solid state. Available studies report that in the case of abovementioned antibiotics, formed degradation products depend on the concentration of investigated drug, pH of the solution, temperature and presence of acids and bases. It has been proved that different products as the result of carbapenem degradation were formed in solid state as well as in aqueous solutions [2]. The main degradation pathway of carbapenems degradation leads to the cracking of β-lactam bond in a bicyclic-fused rings structure. Existing papers have been reported that most of the chromatographic procedures were based on columns filled with 5 μm particles [2–7]. Application of columns with smaller particle sizes was reported for only a few β-lactam antibiotics [8–10]. However, the benefits of such analytical methods can be significant, taking into account higher reduction of the run time of analysis and shorter contact with reagents, which cause the hydrolytic degradation of investigated substances during sample preparation and chromatographic analysis [11, 12]. Therefore, it is such an important study of transfer from HPLC procedure to UPLC separation in the case of labile drugs. The aim of our studies was to select the best analytical conditions for the determination of labile carbapenem analogues, in the presence of their degradation products.

## Experimental

### Standards and Reagents

DORIBAX™ (Janssen-Cilag International NV, Beerse, Belgium) containing doripenem monohydrate as an anhydrous basis form was used as doripenem standard. Meropenem (purity >98 %) was obtained from CHEMOS (Regenstauf, Germany). Tebipenem (purity >98 %) was supplied by Pharmachem International (Wuhan, China). All other chemicals and solvents were obtained from Merck KGaA (Darmstadt, Germany) and were of analytical grade. High-quality pure water was prepared using a Millipore Exil SA 67120 purification system (Millipore, Molsheim, France).

### Instrumentation

The Dionex Ultimate 3000 analytical system consisted of a quaternary pump, an autosampler, a column oven and a diode array detector. Three Kinetex, C18, 100A, 100 × 2.1 mm columns (Phenomenex, Torrance, USA) with different particle sizes (1.7, 2.6 and 5 µm) were tested. The mobile phase composed of acetonitrile and 12 mmol L^−1^ ammonium acetate in different ratios (Table 1). The flow rate of the mobile phase was 1.0 mL min^−1^. The wavelength of the DAD detector was set at 298 nm. Separation was performed at 30 °C.

**Table 1:** HPLC and UHPLC parameters for the determination of selected carbapenems

Chromatography conditions	Doripenem			
	Techniques type			
	HPLC [7]	HPLC	UHPLC	UHPLC
Column type	LiChrospher C-18; 5 µm; fully porous; 250 × 4.6 mm	Kinetex C 18; 5 µm; core shell; 100 × 2.1 mm	Kinetex C 18; 2.6 µm; core shell; 100 × 2.1 mm	Kinetex C 18; 1.7 µm; core shell; 100 × 2.1 mm
Mobile phase	12 mM ammonium acetate–acetonitrile (96:4 v/v)	12 mM ammonium acetate–acetonitrile (96:4 v/v)	12 mM ammonium acetate–acetonitrile (96:4 v/v)	12 mM ammonium acetate–acetonitrile (90:10 v/v)
Flow rate	1.2 mL min^−1^	1.0 mL min^−1^	1.0 mL min^−1^	0.5 mL min^−1^
Detection wavelength	298 nm	298 nm	298 nm	298 nm
*t*_R_	6.2 min	0.823 min	0.869 min	0.666 min
Asymmetry		0.97	1.03	1.38
Pressure		244 bar	822 bar	710 bar
Temperature		30 °C	30 °C	30 °C

Chromatography conditions	Meropenem			
	Techniques type			
	HPLC [4]	HPLC	UHPLC	UHPLC
Column type	LiChrospher C-18; 5 µm; fully porous; 250 × 4.6 mm	Kinetex C 18; 5 µm; core shell; 100 × 2.1 mm	Kinetex C 18; 2.6 µm; core shell; 100 × 2.1 mm	Kinetex C 18; 1.7 µm; core shell; 100 × 2.1 mm
Mobile phase	12 mM ammonium acetate–acetonitrile (92:8 v/v)	12 mM ammonium acetate–acetonitrile (93:7 v/v)	12 mM ammonium acetate–acetonitrile (93:7 v/v)	12 mM ammonium acetate–acetonitrile (90:10 v/v)
Flow rate	1.2 mL min^−1^	1.0 mL min^−1^	1.0 mL min^−1^	0.5 mL min^−1^
Detection wavelength	298 nm	298 nm	298 nm	298 nm
*t*_R_	4.32 min	0.683 min	0.79 min	0.584 min
Asymmetry		0.92	1.12	1.39
Pressure		273 bar	825 bar	710 bar
Temperature		30 °C	30 °C	30 °C

Chromatography conditions	Tebipenem			
	Techniques type			
	HPLC [3]	HPLC	UHPLC	UHPLC
Column type	LiChrospher C-18; 5 µm; fully porous; 250 × 4.6 mm	Kinetex C 18; 5 µm; core shell; 100 × 2.1 mm	Kinetex C 18; 2.6 µm; core shell; 100 × 2.1 mm	Kinetex C 18; 1.7 µm; core shell; 100 × 2.1 mm
Mobile phase	12 mM ammonium acetate–acetonitrile (96:4 v/v)	12 mM ammonium acetate–acetonitrile (93:7 v/v)	12 mM ammonium acetate–acetonitrile (90:10 v/v)	12 mM ammonium acetate–acetonitrile (90:10 v/v)
Flow rate	1.2 mL min^−1^	1.0 mL min^−1^	1.0 mL min^−1^	0.5 mL min^−1^
Detection wavelength	298 nm	298 nm	298 nm	298 nm
*t*_R_	12.32 min	0.87 min	0.533 min	1.16 min
Asymmetry		1.14	1.12	0.76
Pressure		287 bar	825 bar	682 bar
Temperature		30 °C	30 °C	30 °C

### Procedure for Forced Degradation Study of API’s

#### Degradation in Aqueous Solutions

The degradation of analysed API’s (active pharmaceutical ingredient) in aqueous solutions was studied at 293 K in sodium hydroxide solution (0.3 mol L^−1^) and at 353 K in hydrochloric acid (0.3 mol L^−1^). The ionic strength of all solutions was adjusted to 0.5 mol L^−1^ with a solution of sodium chloride (4.0 mol L^−1^). Degradation was initiated by dissolving an accurately weighed 5.0 mg of analysed drug in 10.0 mL of the solution equilibrated to the desired temperature in stoppered flasks. At specified times, samples of the reaction solutions were neutralised and instantly cooled with a mixture of ice and water.

#### Degradation in Solid State

5.0 mg samples of analysed API’s were weighed into 5 mL glass vials. To evaluate the influence of temperature and humidity, the samples were placed in a desiccator containing saturated solution of sodium chloride (RH ~76.4 %) that was in incubators (Wamed, Warsaw, Poland) set to temperature 333 K. At specified time intervals, determined by the rate of degradation, the vials were removed, cooled to room temperature and their contents were dissolved in water. The so-obtained solutions were quantitatively transferred into measuring flasks and diluted with the same solvent to 10.0 mL.

## Results and Discussion

Chromatographic studies of β-lactam analogues are complicated due to their significant susceptibility to degradation, as well as possibility of catalytic effect of their degradation products to stability of parent compound.

In this article, different analytical procedures based on core shell C18 stationary phases with various particle sizes were evaluated. The fully porous C18 columns filled with 5 µm particles allowed the determination of analysed carbapenems in about 15 min [2–8]. Employment of core shell 5 µm particles technology leaded to essential reduction of retention times (*t*_R_) (Table 1). The determination of carbapenem analogues based on the core shell C-18 columns with particles 2.6 µm did not give significant benefits in regard to the reduction of retention times. However, this application produces higher pressures. In the case of determination of carbapenems analogues using core shell C-18 columns with 1.7 µm particles, the retention times represented similar values as in separation based on stationary phase with greater particles, but simultaneously flow rate was twice less (Table 1). This approach is compromised between reduction of consumption organic solvents and high pressure which appears in developed method with 5 µm particles.

**Fig. 1:**
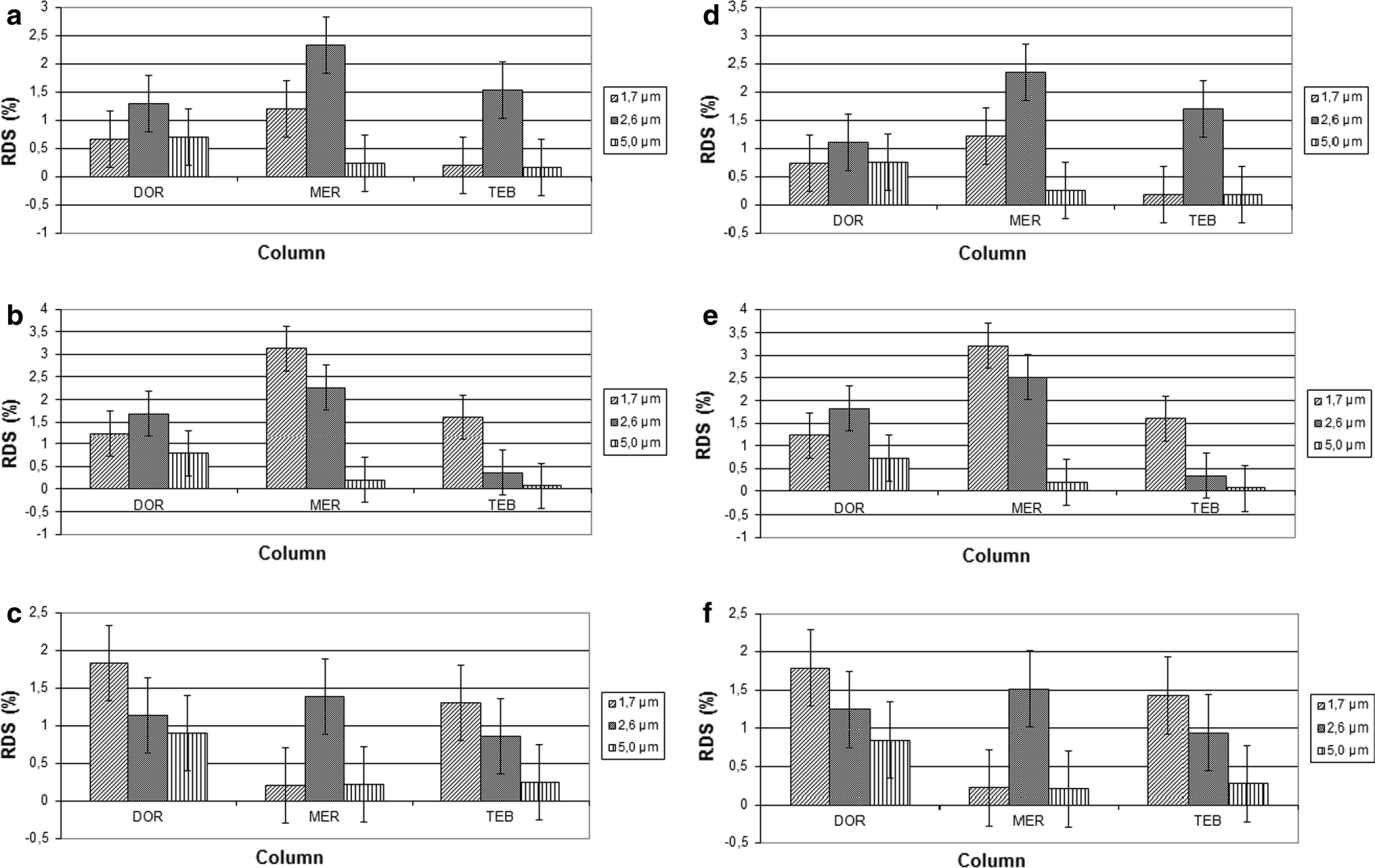
Inter-day (**a**–**c**) and intra-day (**d**–**f**) precision of determination of carbapenem analogues at 80 % (**a**, **d**), 100 % (**b**, **e**) and 120 % (**c**, **f**) levels of initial concentration

Validation of analytical methods for the determination of carbapenems using C-18 stationary phase with different particle sizes was conducted in regarding to linearity, precision, accuracy, LOD and LOQ. The significant reduce *t*_R_ of carbapenem after application of core shell columns did not influence on separation of main peaks (meropenem, doripenem and tebipenem) from degradation products. Suitable symmetry of peaks of main substances as well as related products was unchanged. Order of time retentions for all analytes was the same as in the separation based on fully porous stationary phases. For all the analogues of carbapenems, the linear relationships between concentration of analytes and response of detector were achieved. The widest ranges of linear response were obtained when carbapenems were determined with usage of core shell columns with 5 µm particles. Those determinations were characterised by the most satisfied regression coefficients. Table 2 shows linear response function of concentration over the range 0.5–3.0 µg and regression coefficients (*r*) were >0.995 for all the carbapenems. Similar results were obtained for precision studies. It was shown that the lowest values of intra-day and inter-day precision were calculated in the cases of determination of DOR, MER and TEB using C-18 column filled with 5 µm particles. Method repeatability data as accuracy are summarised in Fig. 1.

**Table 2:** Linear range, regression data, and LODs and LOQs for analysis of selected carbapenems

Column type (µm)	Regression equation	*R* ^2^	Linear range (µg)	LOD (µg)	LOQ (µg)
Doripenem					
1.7	*y* = 39.330*x* + 7.833	0.9954	0.50–3.00	0.12	0.34
2.6	*y* = 20.285*x*	0.9997	0.25–3.00	0.03	0.09
5.0	*y* = 22.324*x*	0.9999	0.05–3.00	0.01	0.03
Meropenem					
1.7	*y* = 36.863*x* + 11.004	0.9928	0.50–3.00	0.14	0.43
2.6	*y* = 19.609*x*	0.9997	0.25–3.00	0.03	0.09
5.0	*y* = 22.314*x*	0.9999	0.05–3.00	0.01	0.02
Tebipenem					
1.7	*y* = 48.147*x*	0.9999	0.50–3.00	0.01	0.03
2.6	*y* = 28.856*x*	0.9999	0.05–3.00	0.01	0.02
5.0	*y* = 22.518*x*	0.9999	0.05–3.00	0.01	0.03

**Fig. 2:**
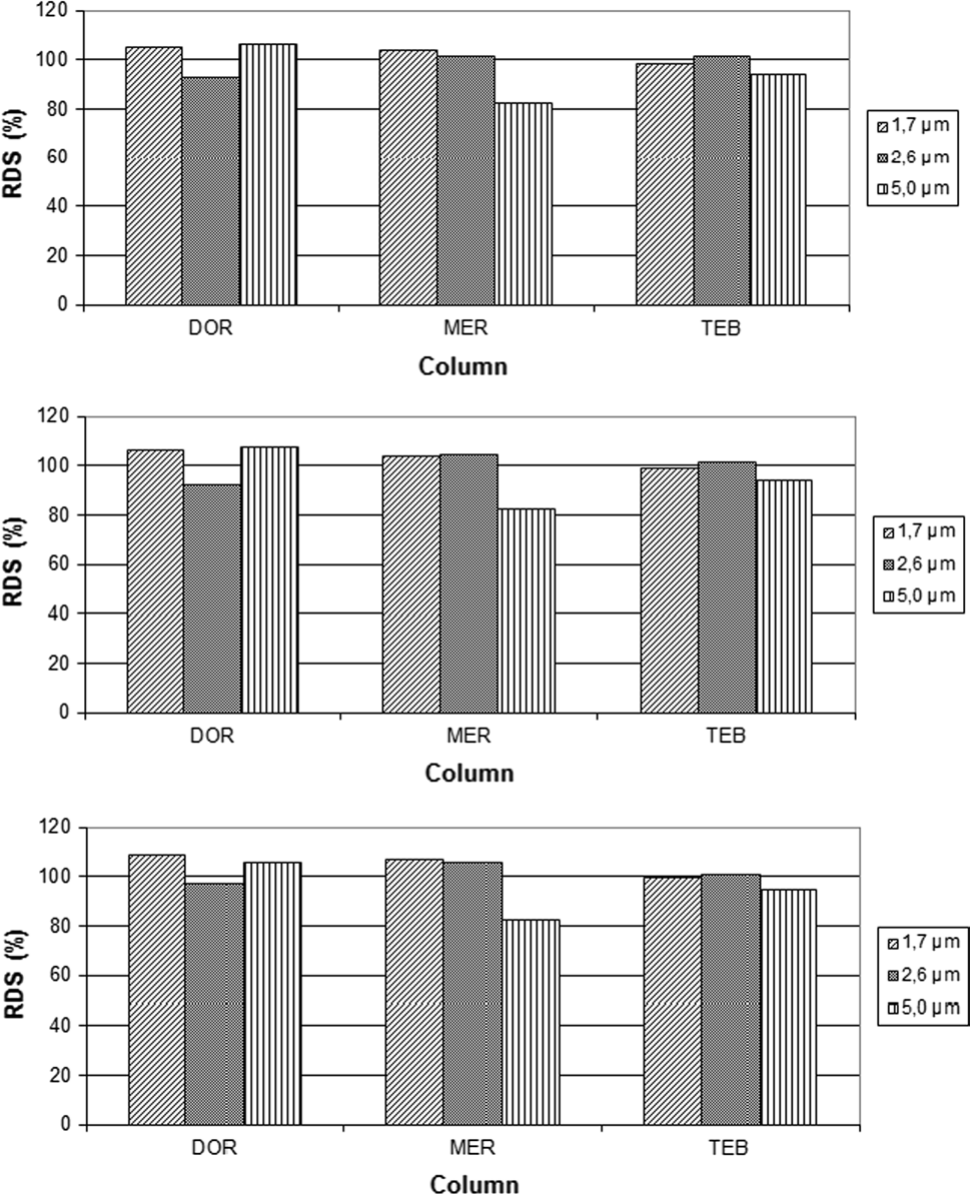
Recovery of determination of carbapenem analogues at 80 % (**a**), 100 % (**b**) and 120 % (**c**) levels of initial concentration

Decreasing particle sizes from 2.6 to 1.7 µm in core shell columns did not significantly influence RDS % values characterising intra-day and inter-day precision. The accuracy of determination of carbapenem analogues was established at three concentration levels: 80,100 and 120 % of label claim. However, significant differences between accuracy of determinations of carbapenems analogues were not observed (Fig. 2a–c). The lowest values of LOD and LOQ for carbapenems were obtained when their determinations were conducted on core shell column filed with 5 µm particles.

## Conclusions

In the presented study, the influence of the particle size of core shell stationary phase on parameters of labile drugs determination was examined. An achievement of the most satisfactory validation parameters was possible when the doripenem, meropenem and tebipenem were determined using core shell C-18, 5 µm stationary phases. The separation of carbapenems based on core shell C-18, 5 µm stationary phases is valuable due to short time of analysis, long vitality of column and possibility to work on classical HPLC systems (dedicated for max pressure 400 bars).
